# Inhibiting Nampt signaling promotes M2 macrophage polarization to enhance bone regeneration in periodontitis

**DOI:** 10.3389/fbioe.2026.1768560

**Published:** 2026-03-27

**Authors:** Yixin Xia, Kexin Xie, Chunhui Liao, Zhanyi Ye, Baojun Long

**Affiliations:** Guangzhou Women and Children’s Medical Center, Guangzhou Medical University, Department of Stomatology, Guangdong Provincial Clinical Research Center for Child Health, Guangzhou, China

**Keywords:** bone regeneration, immune microenvironment, macrophages, NAMPT, periodontitis

## Abstract

**Background:**

Dysregulated macrophage polarization is a fundamental cause of bone loss in periodontitis. However, the role of nicotinamide phosphoribosyltransferase (NAMPT) in macrophage regulation within periodontitis remains unclear. This study aimed to elucidate the function of NAMPT signaling in macrophage polarization and its effects on periodontal bone regeneration.

**Methods:**

Single-cell RNA sequencing (scRNA-seq) data from periodontitis tissues were analyzed to characterize NAMPT^+^ macrophages. Murine macrophages (RAW264.7) were treated with the NAMPT inhibitor FK866 or the enzymatic product of NAMPT and NAD^+^ precursor nicotinamide mononucleotide to assess polarization status and were co-cultured with MC3T3-E1 osteoblasts to evaluate osteogenic potential. The therapeutic effects of local FK866 injection were further examined in a ligature-induced rat periodontitis model using micro-computed tomography (micro-CT), hematoxylin-eosin Staining H&E, Tartrate-resistant acid phosphatase (TRAP), and immunofluorescence staining.

**Results:**

The M2 macrophage subpopulation with low NAMPT expression exhibited strong bone repair–promoting properties. *In vitro*, FK866 treatment reduced M1 polarization and increased M2 polarization, significantly increasing the macrophage-mediated promotion of mineralization. *In vivo*, local FK866 administration decreased osteoclast formation and inflammatory infiltration while promoting alveolar bone regeneration mediated by M2 macrophages.

**Conclusion:**

Inhibition of the NAMPT signaling pathway induces M2 macrophage polarization, alleviating inflammation and facilitating osteogenesis within periodontal bone defects. These findings identify NAMPT as a promising therapeutic target for modulating the host response and promoting bone regeneration in periodontitis.

## 1 Introduction

Periodontitis is a chronic inflammatory disease of the periodontal tissues primarily caused by bacterial infection. Its hallmark feature is the progressive and irreversible resorption of the alveolar bone, which can eventually lead to tooth loss and severely compromise patients' quality of life. Due to its complex pathogenesis, periodontitis remains a major research focus and clinical challenge in the field of oral medicine (Sczepanik et al., 2020). Although conventional periodontal therapies can effectively control bacterial infections and suppress inflammation, achieving functional regeneration of periodontal bone defects remains a significant and unresolved problem. Alveolar bone resorption in periodontitis is essentially caused by the imbalance between the host’s immune-inflammatory response and its intrinsic tissue repair ability. During disease progression, activated immune cells secrete large amounts of pro-inflammatory cytokines, such as interleukin-1β (IL-1β) and tumor necrosis factor-α (TNF-α), which activate osteoclast differentiation and increase bone resorption (Neurath and Kesting, 2024; Ferrara et al., 2025; Papathanasiou et al., 2020). Therefore, identifying strategies to transition the periodontal microenvironment from an inflammatory state to one that supports repair and regeneration is a critical area of current investigation.

To address this challenge, the concept of host regulation therapy has been proposed, aiming to restore immune homeostasis within periodontal bone tissues. This therapeutic strategy aims to disrupt the self-sustaining inflammatory cycle and promote tissue regeneration by regulating immune responses through pharmacological, biological, or material-based interventions (Yang et al., 2021). Among the various immune cell types involved, macrophages play a key role in the complex cellular network that regulates periodontal tissue homeostasis due to their remarkable plasticity and dynamic participation in both inflammation and repair (Mo et al., 2024). Macrophages are broadly classified into two functional phenotypes: pro-inflammatory M1 and pro-regenerative M2 (Lazarov et al., 2023). In the early stages of periodontitis, M1 macrophages are activated to eliminate invading pathogens and release pro-inflammatory mediators to fight against infection. However, excessive or prolonged M1 activation contributes to tissue destruction and alveolar bone loss (Wang et al., 2021; Gao et al., 2023; Li et al., 2024). M2 macrophages exert anti-inflammatory and tissue reparative effects by secreting cytokines such as interleukin-10 (IL-10) and transforming growth factor-β (TGF-β), as well as growth factors including bone morphogenetic protein-2 (BMP2), which directly promote osteogenic differentiation of stem cells and subsequent bone formation (Zhang et al., 2021; Zhang et al., 2024; Yang et al., 2023; Yan et al., 2022).

Multiple studies indicate that the severity of periodontitis is significantly associated with the M1/M2 macrophage ratio within the periodontal immune microenvironment (Zhao et al., 2022; Cui et al., 2023; Chen et al., 2022; Xu et al., 2023). A dominance of M1 macrophages promotes persistent inflammation and inhibits bone regeneration, whereas induction of M2 polarization improves tissue repair. Therefore, therapeutic strategies that actively promote the transition of macrophages from the M1 to the M2 phenotype may represent a more effective approach to achieving periodontal bone regeneration.

Nicotinamide phosphoribosyltransferase (NAMPT) is the rate-limiting enzyme in the nicotinamide adenine dinucleotide (NAD^+^) salvage pathway in mammalian cells, playing a key role in maintaining intracellular NAD^+^ homeostasis (Zu et al., 2025). NAD^+^ serves as a key coenzyme for sirtuins (Sirt) and poly(ADP-ribose) polymerases (PARPs), participating in critical biological processes such as cellular energy metabolism, signal transduction, and inflammatory response (Gasparrini et al., 2021; Gasparrini and Audrito, 2022; Covarrubias et al., 2021). The mechanism by which NAMPT regulates macrophage activity is complex. Activated M1 macrophages rely predominantly on glycolytic metabolism for rapid energy production, which depends heavily on NAMPT-mediated NAD^+^ synthesis. Moreover, M1 macrophages can secrete extracellular NAMPT (eNAMPT) as a damage-associated molecular pattern (DAMP), enhancing inflammatory signaling through activation of the Toll-like receptor 4 (TLR4) pathway (Oka et al., 2023; Hong et al., 2022; Hong et al., 2024). M2 macrophages, which exhibit anti-inflammatory and reparative properties, primarily depend on mitochondrial oxidative phosphorylation, a process that also requires NAMPT to maintain NAD^+^ levels (Liu et al., 2023; Travelli et al., 2018). This dual role of NAMPT in different macrophage subsets presents a biological paradox, making its function as an immunomodulatory target uncertain but scientifically convincing.

Previous research has shown that NAMPT regulates multiple cell types within periodontal tissues. It can activate periodontal ligament fibroblasts and induce their polarization toward pro-inflammatory phenotypes, promoting the release of inflammatory cytokines and matrix metalloproteinases, which exacerbate local inflammation and extracellular matrix degradation (Park et al., 2017). Moreover, NAMPT regulates alveolar bone vascular homeostasis by altering vascular permeability, affecting immune cell recruitment and infiltration (Xiao et al., 2025). It has also been reported to activate osteoclasts, exacerbating alveolar bone resorption and contributing to the progression of periodontal defects (Hassan et al., 2018). These findings underscore the key role of NAMPT in mediating inflammation-driven periodontal destruction. However, its role in regulating macrophage polarization and its subsequent effect on the reparative outcomes of periodontitis-induced bone defects remains to be fully elucidated.

This study aimed to elucidate the regulatory effects of NAMPT inhibition on macrophage polarization and to evaluate its therapeutic potential in the repair of periodontal bone defects. We hypothesize that inhibition of NAMPT may simultaneously reduce inflammation and promote osteogenesis, offering a novel immunomodulatory strategy for host-targeted therapy in periodontitis.

## 2 Methods

### 2.1 Analysis of single-cell RNA sequencing (scRNA-Seq) data

A publicly available scRNA-seq dataset of gingival tissues from patients with periodontitis was obtained from the NCBI Gene Expression Omnibus (GEO) under accession number GSE171213. Data processing and downstream analyses were performed using the Seurat R package (version 5.0). Initial quality control involved exclusion of cells with >20% mitochondrial gene content or <200 and >7,500 detected genes. The filtered data were normalized using the NormalizeData function, and highly variable genes were identified using the FindVariableFeatures function. Dimensionality reduction was conducted via Uniform Manifold Approximation and Projection (UMAP), followed by unsupervised cell clustering. Macrophage populations were identified using the FindMarkers function. Based on NAMPT gene expression levels, macrophages were subdivided into two groups: NAMPT^+^ and NAMPT^−^ subsets. Differential gene expression analysis between these groups was performed using FindMarkers, with significance defined as an absolute log_2_ fold change >0.25 and an adjusted *P* value < 0.05. Functional enrichment analyses, including Gene Ontology (GO), Kyoto Encyclopedia of Genes and Genomes (KEGG), and Gene Set Enrichment Analysis (GSEA), were carried out using the clusterProfiler R package. Cell–cell communication networks were analyzed using the CellChat package.

### 2.2 Cell culture

Murine monocytic macrophage cells (RAW264.7) and murine embryonic osteoblast precursor cells (MC3T3-E1) and primary bone marrow-derived macrophages (BMDM) were obtained from BDBIO (China). RAW264.7 and BMDM cells were maintained in high-glucose Dulbecco’s Modified Eagle Medium (DMEM) supplemented with 10% fetal bovine serum (FBS; Sigma, USA) and 1% penicillin–streptomycin solution (Sigma, USA). MC3T3-E1 cells were cultured in α-Minimum Essential Medium (α-MEM) containing 10% FBS and 1% penicillin–streptomycin. All cells were cultured at 37 °C in a humidified atmosphere containing 5% CO_2_, with media replaced every 2–3 days. Experiments were performed when cell confluence reached approximately 70%–80%.

### 2.3 Cell proliferation

The cells were cultured in distinct conditions for a duration of 24 h. Cellular viability was assessed using CCK-8 reagent (Dojindo, Japan).

### 2.4 NAD^+^ determination

The NAD^+^ contents in RAW264.7 were determined using the NAD^+^/NADH detection kit (Beyotime, China). After 24 h of culture, the cells were treated according to the assay instructions, and the absorbance (OD) at 450 nm was measured using a full-wavelength microplate reader.

### 2.5 Macrophage polarization

For polarization experiments, RAW264.7 cells were seeded in 6-well plates and treated for 24 h with phosphate-buffered saline (PBS; Beyotime, China), dimethyl sulfoxide (DMSO; Sigma, USA), FK866 (10 nM; Sigma, USA), or nicotinamide mononucleotide (NMN; 1 mM; Sigma, USA). FK866 was used as an inhibitor of NAMPT, and NMN was used to activate NAMPT. Following incubation, total RNA was extracted using TRIzol reagent (Thermo Fisher Scientific, USA) and stored at −80 °C for subsequent analyses.

### 2.6 Osteogenic differentiation and staining

Osteogenic differentiation was conducted using a co-culture system with Transwell inserts (Corning, USA) featuring a 0.4 μm pore size. Logarithmically growing MC3T3-E1 cells were seeded into the lower chambers of 24-well plates at a density of 2 × 10^4^ cells/well, while RAW264.7 cells were simultaneously inoculated into the upper Transwell inserts at a density of 5 × 10^4^ cells/well. After cell adhesion, the medium in the upper chambers was replaced with complete culture medium containing one of four treatments: (Sczepanik et al., 2020): PBS, (Neurath and Kesting, 2024), DMSO, (Ferrara et al., 2025), FK866 (10 nM), or (Papathanasiou et al., 2020) NMN (1 mM). The medium in the lower chambers of the 24-well plates was replaced with osteogenic induction media consisting of α-MEM supplemented with 10% FBS, 10 mM β-glycerophosphate, 50 μg/mL ascorbic acid, and 100 nM dexamethasone. The Transwell inserts were subsequently placed into the corresponding wells to initiate co-culture. The medium in both the upper and lower chambers was renewed every 2–3 days throughout the 14-day culture period.

#### 2.6.1 Alkaline phosphatase (ALP) staining

After 7 days of co-culture, the medium was removed, and cells were washed twice with PBS. Cells were then fixed in 4% paraformaldehyde (Beyotime, China) for 15 min, followed by staining using a BCIP/NBT ALP staining kit (Beyotime, China) according to the manufacturer’s instructions.

#### 2.6.2 Alizarin Red S (ARS) staining

After 14 days of co-culture, the medium was removed, the cells were washed with PBS, and then fixed in 4% paraformaldehyde for 15 min. The cells were subsequently stained with a 1% Alizarin Red S solution (pH 4.2; Solarbio, China) for 30 min. For quantitative evaluation of mineralization, stained nodules were dissolved in 10% cetylpyridinium chloride (Sigma, USA), and absorbance was measured at 562 nm.

### 2.7 Quantitative reverse transcription polymerase chain reaction (qRT-PCR)

Total RNA was extracted and reverse-transcribed into cDNA using a reverse transcription kit (Vazyme, China). qRT-PCR was performed using SYBR Green qPCR Master Mix (Vazyme, China) on a LightCycler 480 System (Roche, Switzerland). β-Actin was used as the internal reference gene, and relative gene expression was calculated using the 2^−ΔΔCt^ method. The sequences of all primers used are listed in Supplementary Table S1.

### 2.8 Animal model of periodontitis

All animal procedures were approved by the Ethics Committee of Guangzhou Huateng Biosciences (Ethics Approval Number: C202502-9). Ten male Sprague–Dawley rats (8 weeks old, ∼250 g) were randomly assigned to two primary groups (n = 5 rats/group).

All surgical procedures were conducted under anesthesia. Rats received an intraperitoneal injection of sodium pentobarbital at 50 mg/kg to induce anesthesia. Anesthesia depth was confirmed through the absence of the pedal withdrawal reflex. Experimental periodontitis was induced in rats by ligating the cervical region of both maxillary second M with 5-0 silk sutures. After surgery, local *in situ* injections were administered into the periodontal tissues surrounding the maxillary second M daily for 14 consecutive days. In the first group, rats received PBS (vehicle control) on one side and NMN (200 mg/kg) on the contralateral side. In the second group, rats received DMSO (vehicle control) on one side and FK866 (10 mg/kg) on the opposite side. On day 14, marking the experiment’s conclusion, rats were euthanized *via* intraperitoneal injection of an overdose of sodium pentobarbital (150 mg/kg) following the American Veterinary Medical Association (AVMA) Guidelines for the Euthanasia of Animals (2020). Subsequently, maxillary bone specimens were collected for analysis.

### 2.9 Micro-CT analysis

Excised maxillary specimens were fixed in 4% paraformaldehyde for 48 h and subjected to high-resolution micro-CT using a SCANCO μCT50 scanner (SCANCO, Switzerland). Scanning parameters were set at 74 kV voltage, 114 μA current, and 5 μm resolution. Following image acquisition, three-dimensional reconstructions were performed, and quantitative analyses were conducted using Mimics Research 21.0 software. The primary quantitative parameters included the cementoenamel junction–alveolar bone crest (CEJ–ABC) distance and the bone volume/total volume (BV/TV) ratio to assess alveolar bone resorption and regeneration.

### 2.10 Histological and immunofluorescence analysis

After micro-CT scanning, the maxillary bone specimens were decalcified in 10% EDTA (pH 7.4) for 4 weeks, dehydrated through graded ethanol, cleared in xylene, and embedded in paraffin. Sections were cut at a thickness of 5 μm for subsequent staining procedures.

#### 2.10.1 Hematoxylin and eosin (H&E) staining

Sections were deparaffinized, rehydrated, and stained with hematoxylin and eosin using standard protocols to assess inflammatory cell infiltration in the periodontal tissues.

#### 2.10.2 Tartrate-resistant acid phosphatase (TRAP) staining

Osteoclast activity was visualized and quantified using a TRAP staining kit (Beyotime, China) following the manufacturer’s instructions.

#### 2.10.3 Immunofluorescence staining

Paraffin sections were deparaffinized, rehydrated, and subjected to antigen retrieval in citrate buffer (pH 6.0). Non-specific binding was blocked with 5% bovine serum albumin (BSA) for 1 h at room temperature. Sections were incubated overnight at 4 °C with primary antibodies against NAMPT, iNOS, CD206, BMP2, and RUNX2 (HUABIO, China; 1:200 dilution). After washing with PBS, samples were incubated for 1 h at room temperature in the dark with fluorescent secondary antibodies (Proteintech, China), counterstained with DAPI-containing mounting medium, and visualized under a FV3000 confocal laser scanning microscope (Olympus, Japan). A semi-quantitative analysis of fluorescence intensity was conducted using ImageJ software.

### 2.11 Statistical analysis

All experimental data are presented as mean ± standard deviation (SD). Comparisons between two independent groups were performed using the unpaired Student’s t-test, while comparisons among multiple groups employed one-way analysis of variance (ANOVA) followed by Tukey’s *post hoc* test. Statistical analyses were conducted using GraphPad Prism 9.0 (GraphPad Software, USA). Each experiment was independently repeated at least three times, and differences were considered statistically significant at *P* < 0.05.

## 3 Results

### 3.1 M2 macrophage subpopulation characterized by low NAMPT expression promotes osteoregeneration within periodontal defects

**FIGURE 1:**
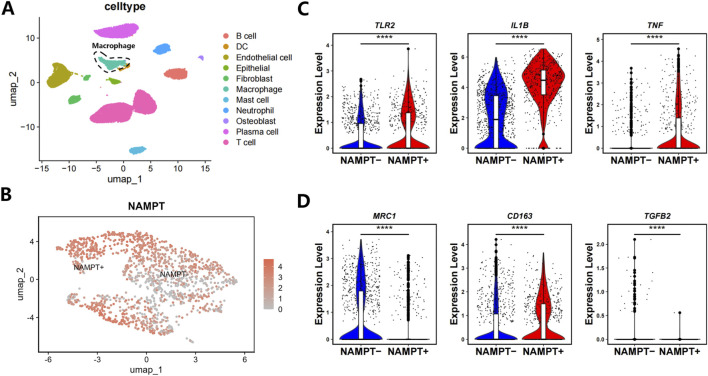
**(A)** UMAP visualization of different cellular populations within the periodontal microenvironment of periodontitis. **(B)** Expression profiles of keygenes in NAMPT^+^ and NAMPT^−^ macrophage subpopulations. **(C)** Expression of M1 macrophage marker genes TLR2, IL1B and TNF in NAMPT^+^ and NAMPT^−^ macrophage subpopulations. **(D)** Expression of M2 macrophage marker genes *MRC1*, *CD163* and *TGFB2* in NAMPT^+^ and NAMPT^−^ macrophage subpopulations.

Single-cell RNA sequencing data from periodontal tissues (GEO dataset GSE171213) were analyzed to elucidate the heterogeneity and functional characteristics of macrophages within the periodontal inflammatory microenvironment. Distinct macrophage subpopulations were identified (Figure 1A) and classified into two major subsets based on NAMPT expression levels: a NAMPT high-expression subpopulation (NAMPT^+^) and a NAMPT low-expression subpopulation (NAMPT^−^) (Figure 1B). Differential gene expression analysis revealed that NAMPT^−^ macrophages exhibited a significant increase in the expression of canonical M2-polarization markers, including *MRC1* (CD206), *CD163*, and *TGFB2*, while the expression of pro-inflammatory genes such as *TLR2*, *IL1B*, and *TNF* was significantly downregulated (Figures 1C,D).

**FIGURE 2:**
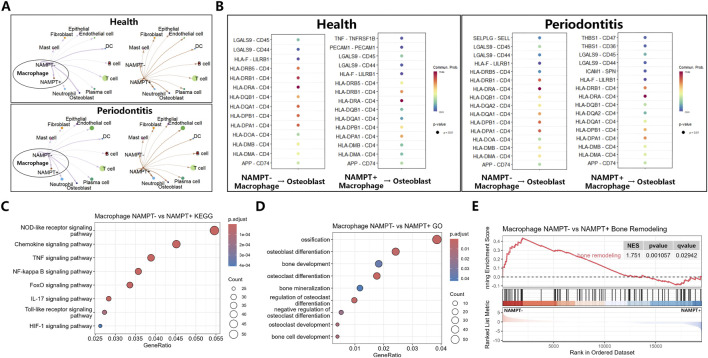
Macrophages with low NAMPT expression enhance the bone-forming ability of osteoblasts. **(A)** Cell communication relationships between the NAMPT-low-expression macrophage subpopulation and other cell subtypes. **(B)** Analysis of cell-to-cell communication signals between NAMPT-low-expressing macrophages and osteoblasts. **(C)** KEGG pathway enrichment analysis. **(D)** GO functional enrichment analysis. **(E)** GSEA enrichment analysis.

Given that NAMPT^−^ macrophages shared transcriptional signatures with the M2 phenotype and exhibited potential pro-reparative functions, the intercellular communication networks between this subpopulation and other cell types in periodontal tissues were evaluated. Cell–cell communication analysis revealed that the NAMPT^−^ macrophage subset displayed particularly stronger interaction signaling with osteoblasts than the NAMPT^+^ subset in both healthy and periodontitis conditions (Figures 2A,B). KEGG pathway enrichment analysis further demonstrated that inflammatory signaling pathways, particularly the NF-κB signaling pathway, were significantly suppressed in NAMPT^−^ macrophages (Figure 2C). GO functional enrichment analysis indicated significant enrichment of tissue-repair–related biological processes, with “skeletal system development” and “osteoblast differentiation” showing the most significant associations (Figure 2D). GSEA confirmed that the NAMPT^−^ macrophage subgroup exhibited increased expression of genes associated with bone remodeling and matrix formation (Figure 2E).

These results demonstrate that reduced NAMPT expression is intrinsically linked to M2-type macrophage polarization and enhanced osteoregenerative capacity within the periodontal microenvironment. Further, targeting NAMPT represents a promising immunoregulatory strategy for shifting macrophage polarization toward the M2 phenotype and promoting periodontal bone regeneration.

### 3.2 *In Vitro* NAMPT inhibition facilitates M2 macrophage polarization and Augments osteogenic modulatory activity

**FIGURE 3:**
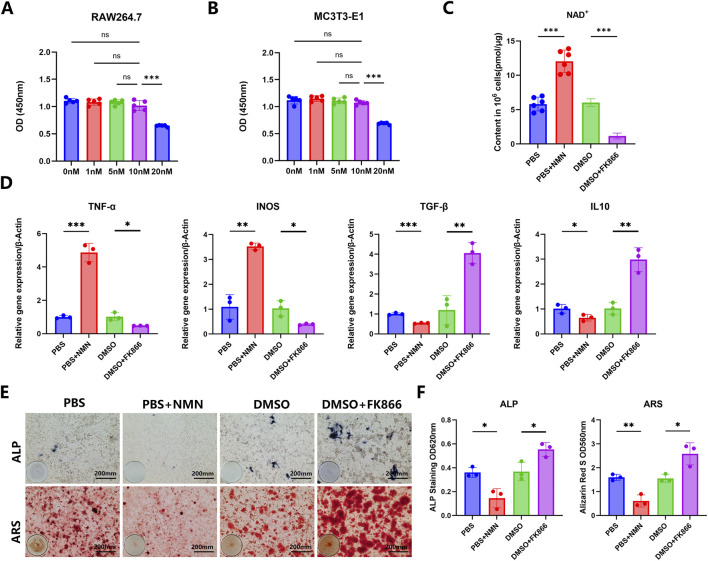
*In vitro* NAMPT inhibition promotes M2 macrophage polarization and enhances osteogenic regulatory ability. **(A)** CCK8 assay after FK866 stimulation of RAW264.7. **(B)** CCK8 assay after FK866 stimulation of MC3T3-E1. **(C)** NAD^+^ detection after stimulation of RAW264.7 with NMN and FK866. **(D)** Effects of FK866 and NMN on the expression of pro-inflammatory cytokines and M2 polarization markers in macrophages. **(E)** In the transwell co-culture model, macrophages treated with FK866 and NMN influence the osteogenic differentiation of osteoblasts through M2 polarization. **(F)** Semi-quantitative analysis of ALP activity and ARS staining. (n = 3; ***P < 0.001, **P < 0.01, *P < 0.05; scale bar = 200 μm).

To elucidate the role of NAMPT signaling in macrophage polarization and its downstream effects on osteogenic regulation, macrophages were treated with the NAMPT inhibitor FK866. This study tested gradient concentrations of FK866 (0nM, 1nM, 5nM, 10nM, 20 nM) to determine its effective concentrations on RAW264.7, MC3T3-E1, and BMDMs. The results showed that the concentration of 10 nM FK866 did not exhibit significant cytotoxicity on these cells (Figures 3A,B; Figure 1A). Therefore, based on the cytotoxicity results, 10 nM FK866 was used in subsequent experiments. Compared to the control group, 10 nM FK866 reduced the NAD^+^ concentration in RAW264.7 cells by about 50%, demonstrating the cellular efficacy at this concentration (Figure 3C). RT-qPCR analysis revealed that FK866 treatment significantly reduced the expression of M1-type polarization markers, including TNF-α and inducible nitric oxide synthase (iNOS), while significantly upregulating M2-type markers, such as TGF-β and IL-10, compared with the DMSO control (Figure 3D). Activation of NAMPT signaling through NMN significantly increased inflammatory cytokine expression, promoting polarization toward the M1 phenotype. To further investigate the functional consequences of NAMPT-mediated macrophage polarization on osteogenic regulation, a co-culture system of macrophages and osteoblasts was established. ALP and ARS staining demonstrated that macrophages pretreated with FK866 showed a significant increase in ALP activity and mineralized nodule formation in osteoblasts, whereas NMN treatment produced the opposite effect, significantly suppressing both osteogenic markers (Figures 3E,F). These findings suggest that inhibiting NAMPT effectively drives macrophage polarization toward the M2 reparative phenotype and improves its paracrine ability to promote osteogenic differentiation *in vitro*.

### 3.3 Inhibition of NAMPT significantly reduced alveolar bone loss in a mouse model of periodontitis

**FIGURE 4:**
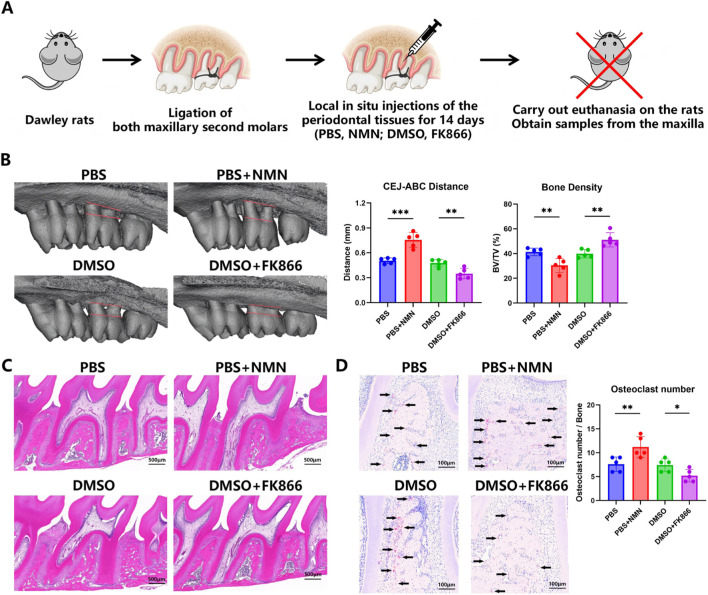
Inhibition of NAMPT significantly ameliorated alveolar bone loss in a murine model of periodontitis **(A)** Flow chart of animal experiment. **(B)** Effects of FK866 and NMN on alveolar bone resorption. **(C)** Effects of FK866 and NMN on periodontal tissue inflammation. **(D)** Effects of FK866 and NMN on osteoclast numbers. (n = 5; ***P < 0.001, **P < 0.01, *P < 0.05; scale bars = 500 μm, 200 μm).

To evaluate the regulatory effects of NAMPT signaling *in vivo*, a rat model of periodontitis was established via periodontal ligation (Figure 4A), followed by local injections of FK866 or NMN, with DMSO and PBS serving as respective controls. Micro-CT three-dimensional reconstruction and quantitative analysis revealed that the CEJ–ABC distance was significantly reduced in the FK866-treated group compared with the DMSO group, while the BV/TV was significantly increased. These results indicate that FK866 treatment effectively mitigated alveolar bone resorption and promoted bone regeneration. Local NMN administration exacerbated bone loss, as shown by an increased CEJ–ABC distance and a decrease in BV/TV (Figure 4B). Histological analyses further supported these findings. H&E staining demonstrated a significant reduction in inflammatory cell infiltration within the periodontal membrane following FK866 treatment (Figure 4C). Moreover, TRAP staining showed a significant decline in osteoclast numbers in the FK866 group relative to DMSO controls, while NMN administration significantly elevated osteoclast numbers compared with the PBS group (Figure 4D). These *in vivo* findings demonstrate that NAMPT inhibition alleviates inflammation, suppresses osteoclast-mediated bone resorption, and promotes alveolar bone remodeling and regeneration in periodontitis, supporting its potential as a therapeutic target for host modulation therapy.

### 3.4 NAMPT-facilitated immune microenvironment reconfiguration improves periodontal bone regeneration

**FIGURE 5:**
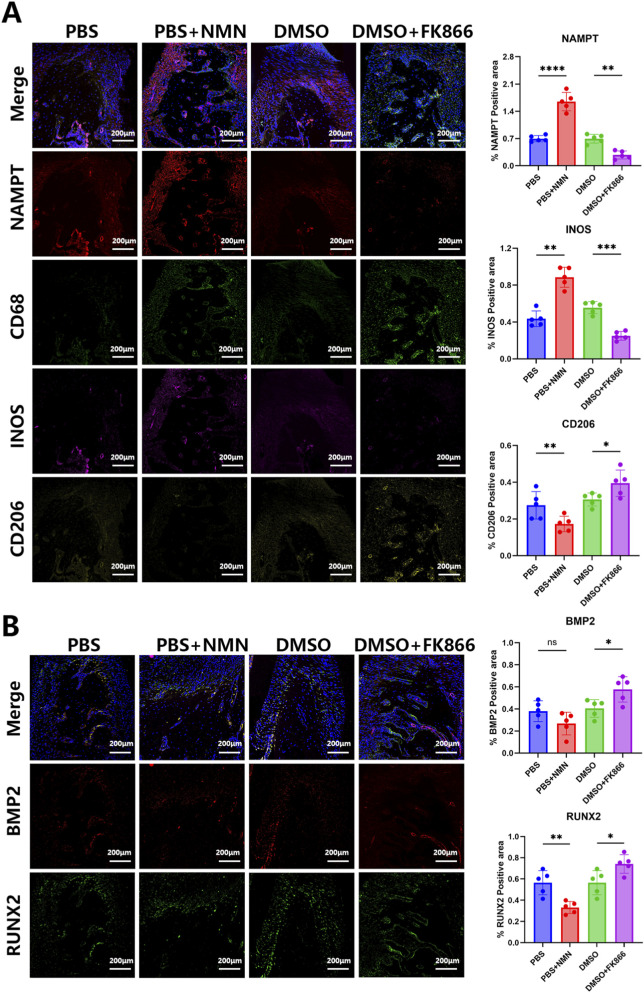
NAMPT-mediated reprogramming of the immune microenvironment promotes periodontal bone regeneration. **(A)** Effects of FK866 and NMN on macrophage polarization-associated protein expression in periodontal tissues. **(B)** Effects of FK866 and NMN on osteogenesis-related protein expression in periodontal tissues. (n = 5; ***P < 0.001, **P < 0.01, *P < 0.05; scale bar = 200 μm).

To elucidate the immunomodulatory mechanism by which FK866 promotes alveolar bone repair, immunofluorescence staining was performed on periodontal tissue sections from the experimental periodontitis model. The fluorescence results revealed that both NAMPT and the M1 macrophage marker iNOS were significantly downregulated in the periodontal tissues of the FK866-treated group compared with the DMSO control, whereas expression of the M2 macrophage marker CD206 was significantly elevated (Figure 5A). These findings indicate that FK866 effectively induces macrophage transition from the pro-inflammatory M1 phenotype to the pro-reparative M2 phenotype within the periodontal bone immune microenvironment. Further analysis of osteogenesis-associated proteins demonstrated that FK866 treatment significantly increased the number of osteoblasts and enhanced the expression of BMP2 and Runt-related transcription factor 2 (RUNX2) compared with the control group (Figure 5B). NMN administration, on the other hand, led to elevated expression of NAMPT and iNOS, accompanied by decreased CD206, BMP2, and RUNX2 levels, suggesting activation of the M1 pro-inflammatory phenotype and aggravation of bone defects. These results demonstrate that inhibition of NAMPT signaling reshapes the periodontal immune microenvironment by promoting macrophage polarization toward the M2 phenotype, enhancing osteogenic signaling pathways, and facilitating periodontal bone regeneration. This immunoregulatory mechanism highlights the therapeutic potential of targeting NAMPT to achieve functional bone repair in periodontitis.

## 4 Discussion

This study elucidated the regulatory role of NAMPT in macrophage-mediated bone repair and its involvement in the regeneration of periodontal bone defects. Our findings demonstrate that inhibition of NAMPT signaling transforms the local periodontal immune microenvironment from a pro-inflammatory and destructive state to a pro-repair and regenerative one. This effect involves the suppression of M1 macrophage–mediated inflammatory cascades and the facilitation of M2 macrophage polarization, increasing osteogenic regulatory activity. These data suggest that NAMPT may serve as a promising therapeutic target for periodontitis, offering a novel mechanistic basis for the development of anti-inflammatory and pro-regenerative interventions in periodontal therapy.

NAMPT is the rate-limiting enzyme in the mammalian NAD^+^ salvage pathway, which maintains intracellular NAD^+^ concentrations and regulates various cellular processes (Wang et al., 2021; Gao et al., 2023; Li et al., 2024). Therefore, its biological significance has gained significant research interest across various fields, including aging, oncology, and immunology. In aging research, decreased NAMPT activity and the resulting decline in NAD^+^ are recognized as key drivers of age-associated degenerative disorders (van der Veer et al., 2007; Song et al., 2015; Li et al., 2022). In cancer biology, tumor cells often exhibit increased dependence on NAMPT to sustain their aberrant proliferation and metabolic activity, making NAMPT inhibitors potential anti-cancer therapeutics (Nacarelli et al., 2020; Lin et al., 2015; Narayanan et al., 2025). In inflammation-related studies, NAMPT has been shown to exert significant pro-inflammatory effects in systemic inflammatory diseases, including rheumatoid arthritis (Ye et al., 2024; Zhou et al., 2022) and atherosclerosis (Opstad et al., 2023). NAMPT regulates the differentiation, activation, and survival of immune cells such as T cells and macrophages via NAD^+^-dependent signaling pathways (Garcia et al., 2022; Sun et al., 2023). Furthermore, NAMPT can induce inflammatory responses by activating the TLR4 signaling pathway and maintaining elevated glycolytic activity in M1 macrophages (Zhou et al., 2022). These findings underscore that NAMPT inhibition may represent an effective strategy for reducing inflammation (Semerena et al., 2023).

Although the pro-inflammatory roles of NAMPT have been extensively characterized in systemic inflammatory and tumor immune contexts, its immunomodulatory functions in the periodontal bone defect microenvironment remain poorly defined. This study reveals that NAMPT inhibition in periodontitis significantly reduces the abundance of pro-inflammatory M1 macrophages and osteoclasts, while promoting the accumulation of reparative M2 macrophages simultaneously. This regulatory pattern differs from earlier approaches that relied on exogenous cytokines, such as interleukin-4 (IL-4) and interleukin-13 (IL-13), to induce M2 polarization (Luo et al., 2024). By targeting the multifunctional endogenous regulator NAMPT, this strategy offers a more intrinsic and sustainable mechanism for changing the direction of macrophage polarization in periodontitis.

The results of this study provide a new perspective for advancing host modulation therapy in periodontitis. Current host-modulating agents, such as doxycycline, primarily function by inhibiting matrix metalloproteinase (MMP) activity to prevent tissue degradation, but their ability to promote tissue regeneration remains limited (Hajishengallis et al., 2020; Okić Đorđević et al., 2023; Golub et al., 1995; Kormi et al., 2014). In recent years, macrophage polarization has emerged as a key focus in regenerative medicine, as directing macrophages toward reparative phenotypes can enhance tissue repair and regeneration. Numerous studies have reported that small-molecule compounds or bioactive scaffolds can regulate macrophage polarization, promoting the regeneration of bone, cartilage, and myocardial tissue (Rodríguez-Morales and Franklin, 2023). However, achieving precise regulation of macrophage polarization within the complex bone immune microenvironment of periodontitis remains a significant challenge (Mo et al., 2024). This study demonstrated that the small-molecule inhibitor FK866 effectively promotes macrophage polarization toward the M2 phenotype and increases their osteoregulatory function by inhibiting NAMPT expression within the periodontal bone defect microenvironment. This inhibition was accompanied by a significant upregulation of BMP2 and RUNX2 expression in osteoblasts, indicating higher osteogenic signaling. These findings suggest that NAMPT inhibition establishes a mechanistic link between immune remodeling and bone regeneration in periodontitis, improving the feasibility and therapeutic potential of host modulation–based treatment strategies.

The underlying molecular mechanism can be explained through immunometabolic regulation. Previous studies have established that M1 macrophages mainly rely on glycolysis, whereas M2 macrophages depend mainly on mitochondrial oxidative phosphorylation (OXPHOS) for energy production (Gao et al., 2023; Li et al., 2024). We hypothesize that glycolysis-activated M1 macrophages exhibit higher dependence on the NAMPT–NAD^+^ axis during the early inflammatory phase of periodontitis. Thus, FK866-mediated NAMPT inhibition likely decreases intracellular NAD^+^ levels, selectively inhibiting the metabolic activity and function of M1 macrophages. This reduction triggers an anti-inflammatory response, decreasing M1 polarization. The resulting metabolic stress may induce metabolic reprogramming, favoring a shift toward an OXPHOS-dependent phenotype that facilitates M2 macrophage polarization. However, because FK866-mediated NAD^+^ depletion also reduces OXPHOS activity, other signaling mechanisms may be involved in this transition and warrant further investigation.

More importantly, the potential biological toxicity of FK866 remains an issue that cannot be ignored. In this study, we found that the local application of FK866 at a concentration of 10 nM in the periodontal bone defect area can effectively regulate the periodontal microenvironment by inhibiting NAMPT, thereby polarizing macrophages towards the M2 phenotype and promoting bone regeneration. The concentration of 10 nM FK866 is generally consistent with the concentrations used in current research in the field of bone defects (Li et al., 2013; He et al., 2017; He et al., 2021), which also partially explains why the application of this concentration in our cell experiments is reasonable. Current related research on FK866 mainly focuses on the preclinical stage, including animal experiments and *in vitro* model studies. In mouse xenograft models and patient-derived organoid models, Sauriol et al. found that FK866 at a concentration of 10 nM, as a NAMPT inhibitor, has a synergistic anti-tumor effect when combined with Olaparib, which can be used to combat the acquired and inherent resistance of ovarian cancer to PARP inhibitors. The mechanism is that the combination therapy significantly depletes intracellular NAD^+^ levels, induces DNA double-strand breaks, and promotes apoptosis (Sauriol et al., 2023). In preclinical animal models, Nahimana and Park et al. found that to achieve a significant inhibition of tumor growth, the commonly used dose is 30 mg/kg (Nahimana et al., 2009; Park et al., 2022). In early clinical studies, Holen et al. found that Daporinad is mainly administered as a single-agent anti-cancer drug through continuous intravenous infusion, with a recommended dose of 0.126 mg/m^2^/h, and its efficacy is limited by systemic diseases such as thrombocytopenia (Holen et al., 2008). In phase I/II clinical studies (NCT00435084) and phase II multicenter open-label studies (NCT00431912), it was found that the recommended dose of FK866 for treating B-cell chronic lymphocytic leukemia and cutaneous T-cell lymphoma is 0.126 mg/m^2^/hr, and none of the above studies reported overall NAD^+^ depletion in cells, animals, or humans.

The clinical translation of FK866 for the treatment of periodontitis faces some inherent limitations. Although FK866 can induce macrophages to polarize from pro-inflammatory M1 type to anti-inflammatory repair M2 type by regulating metabolic remodeling in specific environments, thereby theoretically constructing an immune microenvironment conducive to tissue regeneration, when the concentration of FK866 exceeds a certain threshold, its inhibition of cellular energy metabolism directly affects osteoprogenitor cells (He et al., 2017). This high-intensity metabolic interference may inhibit the expression of key osteogenic genes such as Runx2 and OCN, leading to impaired mineralization capacity of bone tissue. Therefore, although M2 macrophages release some pro-repair factors, the osteoblasts themselves are in a metabolically restricted state and cannot effectively respond to these repair signals, ultimately resulting in the observed inhibition of osteogenic effects in experiments. The application effect of FK866 in the treatment of periodontitis depends on the balance between inducing anti-inflammatory polarization and maintaining osteoblast metabolism. If the concentration cannot be precisely controlled, its direct osteogenic inhibitory effect will dominate, leading to a decrease in bone regeneration effects, presenting biological manifestations contrary to the initial intention of immune regulation. Although the local dose used in this study is low, the narrow therapeutic index of NAMPT inhibitors remains concerning. While local administration can minimize systemic exposure, the risk of affecting high metabolic tissues such as hematopoietic cells must be strictly assessed through long-term safety studies. Additionally, the bioavailability and retention of FK866 in the dynamic periodontal environment pose significant pharmacological challenges. Future studies should explore controlled-release carriers, such as hydrogels or functionalized scaffolds, to maintain the drug at an optimal concentration of 10 nM. Furthermore, the potential activation of the salvage pathway mediated by NAPRT in inflammatory tissues may lead to metabolic bypasses, thereby reducing the long-term efficacy of the drug in chronic inflammatory environments. Recognizing these obstacles is crucial for refining FK866-based dental regeneration strategies.

This study identifies NAMPT as a key molecular link between immune regulation and bone regeneration in periodontitis, highlighting the therapeutic potential of targeting this pathway. By demonstrating that NAMPT inhibition simultaneously exerts anti-inflammatory and pro-osteogenic effects, this work introduces a dual-function therapeutic target for periodontitis. Future research should focus on developing localized sustained-release formulations targeting NAMPT and systematically evaluating their effects in other inflammatory bone loss diseases, which could provide valuable insights for translational and clinical applications.

## 5 Conclusion

This study confirms that inhibition of NAMPT promotes the transition of macrophages from the M1 pro-inflammatory phenotype to the M2 reparative phenotype, achieving dual outcomes: a reduction in inflammation and an enhancement of bone regeneration within the periodontal bone defect microenvironment. These findings identify NAMPT as a promising therapeutic target for periodontitis, providing a mechanistic foundation for developing novel drugs that integrate immune modulation with regenerative potential.

## 6 A one-sentence summary

NAMPT as a promising therapeutic target for modulating the host response and promoting bone regeneration in periodontitis.

## Data Availability

The datasets presented in this study can be found in online repositories. The names of the repository/repositories and accession number(s) can be found in the article/Supplementary Material.

## Supplementary material

The Supplementary Material for this article can be found online at: https://www.frontiersin.org/articles/10.3389/fbioe.2026.1768560/full#supplementary-material

SUPPLEMENTARY TABLE S1 Primer sequences for target and reference genes.

SUPPLEMENTARY FIGURE S1
*In vitro* NAMPT inhibition promotes M2 macrophage polarization of BMDM. **(A)** CCK8 assay after FK866 stimulation of BMDM. **(B)** Effects of FK866 and NMN on the expression of pro-inflammatory cytokines and M2 polarization markers in BMDM.
