# Supplementary material for: Inhibiting Nampt signaling promotes M2 macrophage polarization to enhance bone regeneration in periodontitis
Source: Front Bioeng Biotechnol. 2026 Mar 27;14:1768560. doi: 10.3389/fbioe.2026.1768560 (PMC13066245; doi:10.3389/fbioe.2026.1768560)
Supplement: Supplementary file 1 [file Supplementaryfile1.docx]

Supplementary Figure 1


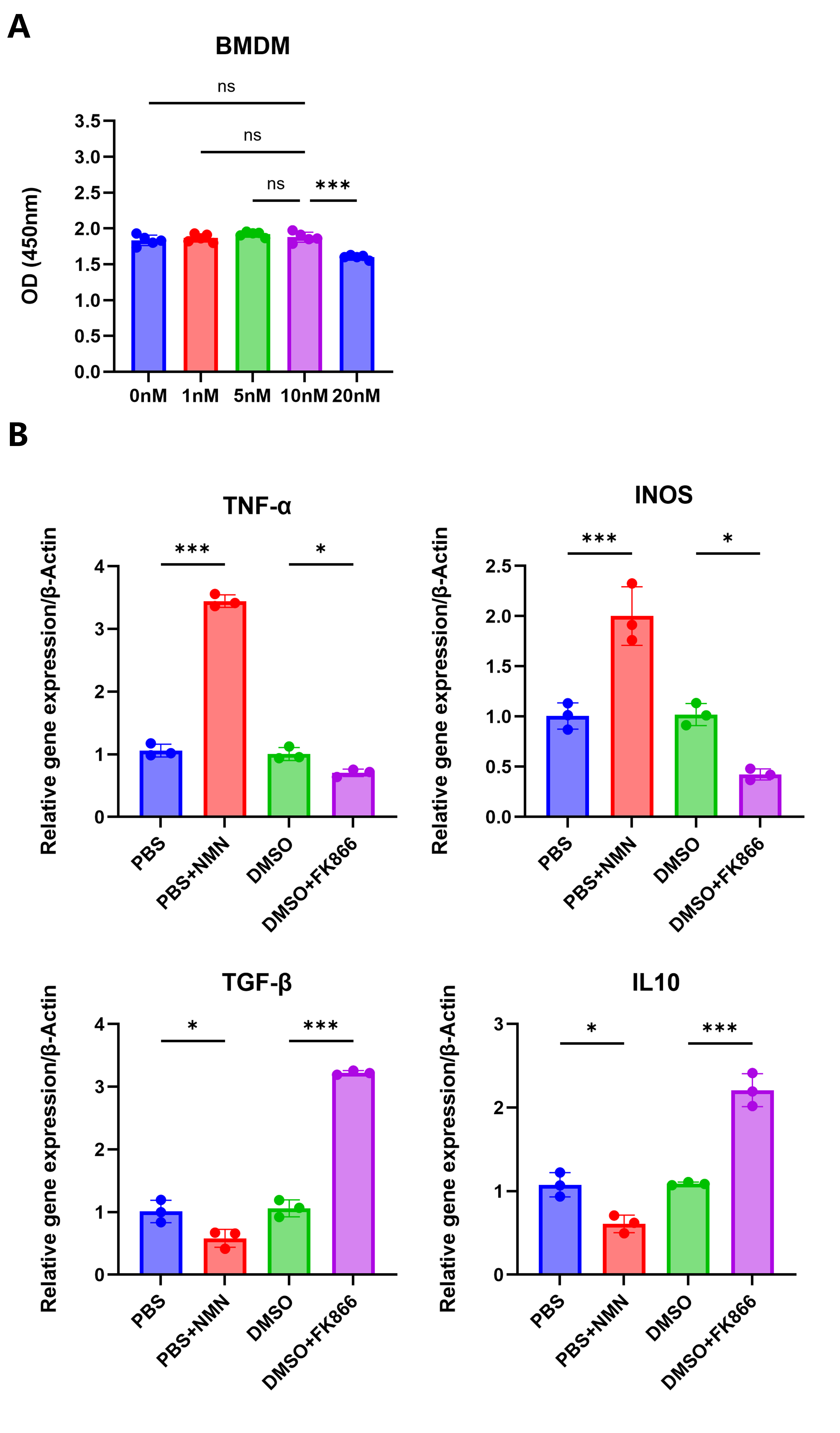


Figure S1. *In vitro* NAMPT inhibition promotes M2 macrophage polarization of BMDM .
(A) CCK8 assay after FK866 stimulation of BMDM. (B) Effects of FK866 and NMN on the expression of pro-inflammatory cytokines and M2 polarization markers in BMDM.
