# Supplementary material for: Inhibiting Nampt signaling promotes M2 macrophage polarization to enhance bone regeneration in periodontitis
Source: Front Bioeng Biotechnol. 2026 Mar 27;14:1768560. doi: 10.3389/fbioe.2026.1768560 (PMC13066245; doi:10.3389/fbioe.2026.1768560)
Supplement: Supplementary file 2 [file Table1.docx]

Supplementary Table 1

**Table S1.** Primer sequences for target and reference genes

| Gene | Forward/Reverse | Sequence (5′-3′) |
| --- | --- | --- |
| *TNF-α* | Forward | CCCTCACACTCAGATCATCTTCT |
|  | Reverse | GCTACGACGTGGGCTACAG |
| *INOS* | Forward | GTTCTCAGCCCAACAATACAAGA |
|  | Reverse | GTGGACGGGTCGATGTCAC |
| *TGF-β* | Forward | CCGCTGCATATCGTCCTGTG |
|  | Reverse | AGTGGATGGATGGTCCTATTACA |
| *IL-10* | Forward | GCTCTTACTGACTGGCATGAG |
|  | Reverse | CGCAGCTCTAGGAGCATGTG |
